# Norepinephrine-functionalised nanoflower-like organic silica as a new adsorbent for effective Pb(II) removal from aqueous solutions

**DOI:** 10.1038/s41598-018-36644-1

**Published:** 2019-01-22

**Authors:** Junkai Gao, Xiuwang Guo, Wenwen Tao, Dian Chen, Jinshu Lu, Yan Chen

**Affiliations:** School of Port and Transportation Engineering, Zhejiang Ocean University, Zhoushan, 316022 China

## Abstract

In order to remove Pb(II) ions efficiently from aqueous solutions, a new effective adsorbent of norepinephrine-functionalised nanoflower-like organic silica (NE-NFOS) was synthesised by a biomimetic method. Biomimetic functionalization with norepinephrine has the advantages of environment-friendly and easy operation. Characterization of the NE-NFOS using scanning electron microscopy, transmission electron microscopy, Brunauer-Emmett-Teller method, and Fourier-transform infrared spectroscopy revealed that the NFOS was modified successfully by norepinephrine. Furthermore, the influences of different parameters including adsorption kinetics, solution pH, adsorption isotherms, concentrations of Na^+^, K^+^, Ca^2+^, and Mg^2+^, desorption and reusability were studied. The adsorption experiments showed that the capacity of NE-NFOS to adsorb Pb(II) ions improved greatly after functionalisation and adsorption equilibrium was attained within 90 min at a pH of 6.0. The Na^+^, K^+^, Ca^2+^, and Mg^2+^ concentrations had little influence on the adsorption, and after recycling for five times, the Pb(II) ion removal efficiency of the adsorbent was more than 79% of its initial value. Thus, it was demonstrated that the NE-NFOS with excellent adsorption performance could be a suitable adsorbent for Pb(II) ions removal in practical applications.

## Introduction

Lead(II) ions are among the most harmful and plentiful of heavy metal ions. Lead(II) ions and compounds find extensive usage in industrial activities such as steel making, chemical production, and metallurgical mining^1–3^. However, massive amounts of Pb(II) ions are inevitably discharged into aqueous solutions and cause pollution. Because of their potential non-biodegradability and bioaccumulation, Pb(II) ions pose a severe threat to the environment and human health^4,5^. Therefore, it is imperative that these heavy metal ions should be removed from water. Although several methods are used to remove Pb(II) ions, including catalytic reduction, adsorption, electrochemical reduction, and ion-exchange^6–9^, adsorption is considered to be the most economical and promising approach because of availability of materials with high adsorption capacities, low cost, and ease of operation^10^. Activated carbon, silica, and polymer resins serve as adsorbent materials and are used in a wide range of applications^11–13^. Mesoporous silica has attracted remarkable attention as an adsorbent material owing to its simple synthetic process, large surface area and pore volume, and ease of surface modification^14,15^.

However, the application of mesoporous silica in the removal of heavy metal ions has been limited by its relatively low adsorption capacity^16–18^. Some functional groups including thiol, amine and carboxyl can be grafted on the surface of mesoporous materials to improve their capacity to adsorb heavy metal ions. Shahbazi *et al*.^16^ studied SBA-15 mesoporous silica functionalised with melamine-based dendrimer amines to remove heavy metal ions. The adsorption results indicated that the dendrimer amine ligands on the adsorbent’s surface enhanced its binding affinity with Pb(II), Cu(II), and Cd(II) ions. Yuan *et al*.^17^ synthesised a novel amino-functionalised microsphere, composed of a mesoporous silica shell and magnetic core, which could enhance its capacity to adsorb heavy metal ions such as Pb(II), Cu (II), and Cd(II). Zhang *et al*.^18^ researched amine-functionalised carbon nanotubes to remove Cu(II) ions. The amine groups on the surface of the adsorbent could form coordination compounds with Cu(II) ions and thus improved the adsorption ability significantly. He *et al*.^19^ synthesized mesoporous silica-calcium phosphate (MS-CP) hybrid nanoparticles as the adsorbent for Cd(II) removal from aqueous solution. The maximum adsorption capacity of Cd(II) by MS-CP was above 153 mg/L, which was ascribed to the electrostatic interaction between the Cd(II) and silanol groups on the surface of MS-CP and ion-exchange between the Cd(II) and calcium in MS-CP.

However, traditional modification strategies have some shortcomings such as complex operation, high energy consumption, and complex involvement of hazardous reagents^20^. In recent years, biomimetic functionalization for mesoporous materials with dopamine have attracted extensive attention as they are inexpensive, adhesive, and possess excellent adsorption efficiencies^21–24^. Gao *et al*.^21^ synthesised graphene hydrogel functionalised with polydopamine for water purification. The presence of abundant functional groups of polydopamine and the high specific surface areas of graphene hydrogel contributed to high capacities for wastewater adsorption. Chen *et al*.^22^ prepared dopamine-functionalised meso-structured silica (MOS) to remove Cd(II) ions, and its adsorption capacity improved evidently over that of meso-structured silica. Gao *et al*.^23^ reported an effective approach for utilising dopamine-functionalised mesoporous silica nanoparticles (Dop-TMSNs) to remove Cu(II) ions; Dop-TMSNs exhibited high performance in the adsorption of Cu(II) ions. Zhu *et al*.^24^ synthesized a novel adsorbent through waste paper derived carbon that was coated with polydopamine, and its hierarchically interconnected porous structure and large specific surface area contributed to the excellent absorption capacity for uranium (VI) in simulated seawater.

The molecule of norepinephrine, which also contains the catechol and amino groups, is similar in structure to dopamine. This suggests that norepinephrine might also exhibit the ability of binding with heavy metal ions. Additionally, biomimetic functionalization with norepinephrine has the advantages of environment-friendly and easy operation^25^. However, norepinephrine has not been used to date as a surface functionalisation reagent in the adsorption of metal ions. Therefore, ongoing efforts are still required for the studies of norepinephrine-functionalised mesoporous silica as the adsorbent to remove heavy metal ions from aqueous solutions.

Nanoflower-like organic silica (NFOS) has attracted great attention in recent years owing to its high porous ratio, large specific surface area, and great mechanical strength^26,27^. Moreover, the NFOS has wrinkled channels, a structure that has the potential for preventing the leakage of modifier, and then the amount of modifier grafted on the surface of NFOS could be increased, which was beneficial for increasing its adsorption capacity for heavy metal ions. However, there are no reports thus far in the literature investigating the application of NFOS to remove metal ions.

In this study, norepinephrine-functionalised nanoflower-like organic silica (NE-NFOS) with a large surface-to-volume ratio was synthesised and firstly applied as an adsorbent to remove Pb(II) ions. The influence of different parameters including adsorption kinetics, solution pH, adsorption isotherms, concentrations of Na^+^, K^+^, Ca^2+^, and Mg^2+^, desorption, and reusability on the process of adsorption was studied, and the adsorption mechanism between the Pb(II) and the NE-NFOS was elucidated. Moreover, for the reason that the functional groups of phenolic and amino ligands in the molecules of norepinephrine have the ability of binding a range of metal ions^28^, the adsorption capacities of NE-NFOS for Cd(II) and Cu(II) ions were also evaluated.

## Experimental section

### Materials

Norepinephrine was purchased from Kangbaotai Fine-chemicals Co., Ltd, China. Cetyltrimethyl ammonium bromide (CTAB) and 1,2-Bis(triethoxysilyl)ethane (BTSE) were purchased from Aldrich-Sigma and Shanghai Macklin Biochemical Co., Ltd, respectively. All other reagents, which were of analytical grade, were obtained from Sinopharm Chemical Reagent Co., Ltd, China. Lead nitrate (Pb(NO3)_2_) was used to prepare a standard stock solution of Pb(II) ions in deionised water. Then, the stock solution was then diluted to obtain Pb(II) ion concentration of 10~100 mg/L.

### Preparation of nanoflower-like organic silica (NFOS)

The method for synthesising the NFOS was modified according to a previous study^29^. First, 1.25 g of CTAB, 1.25 g of n-butanol, and 5 g of cyclohexane were added to 100 g of 0.4 M aqueous urea solution, and the mixture was stirred for 30 min. Next, 0.875 g of tetraethyl orthosilicate and 0.375 g of BTSE were added together to the solution, and the resulting suspension was stirred for 30 min at room temperature (25 ± 1 °C). Moreover, the solution was maintained at 70 °C for another 24 h, following which the solid component was filtered out and washed thrice with ethanol and deionised water. Next, 250 mL of acetone was slowly added to the mixture and refluxed at 80 °C for 48 h to remove the template. The product was then washed with ethanol several times and dried at 45 °C for 24 h. The NFOS was thus obtained.

### Preparation of norepinephrine-functionalised NFOS (NE-NFOS)

The NFOS was functionalised with norepinephrine by the post-grafting method. Specifically, 0.5 g of NFOS was added into 100 mL of 1 g/L norepinephrine solution freshly prepared in a phosphate buffer (pH 8.5), and the mixture was stirred for 3 h. Next, the solid product was filtered, washed with distilled water several times, and dried at 40 °C for 24 h. The NE-NFOS was thus obtained.

### Batch adsorption experiments

**Figure 1:**
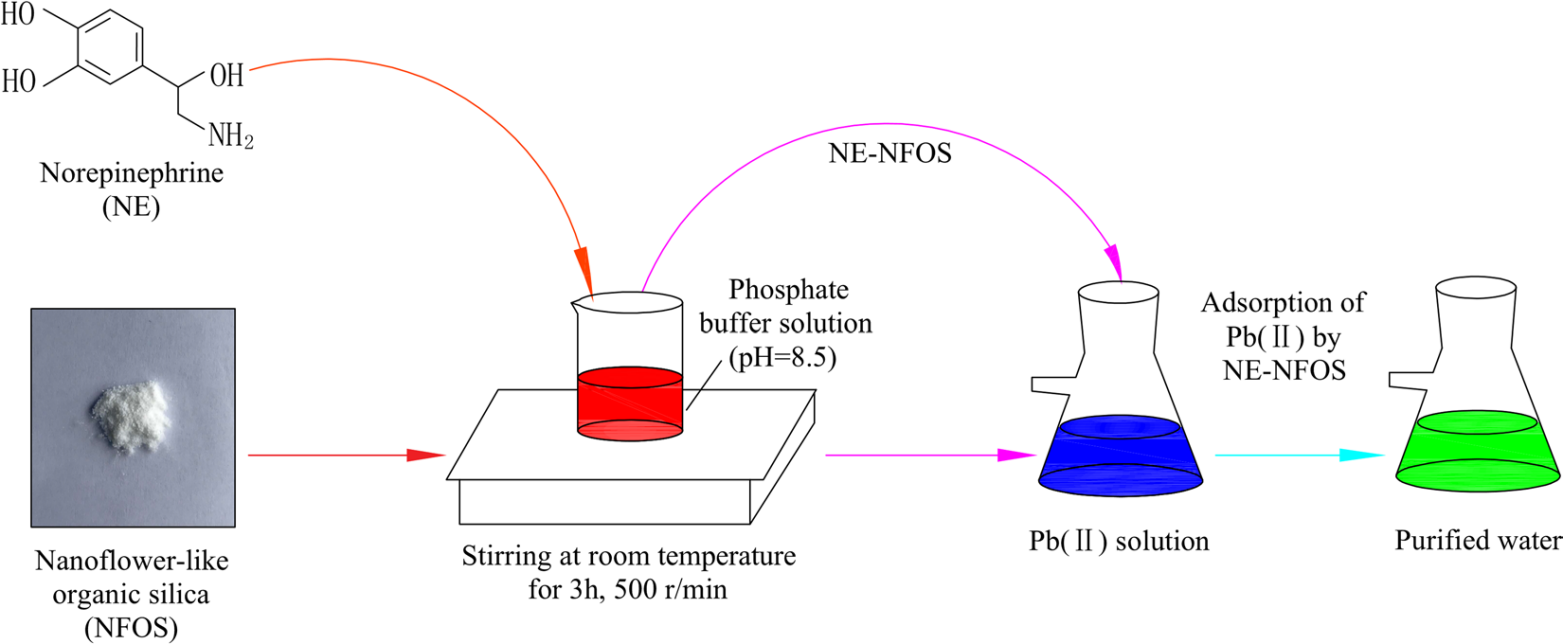
Schematic illustration of NE-NFOS preparation and Pb(II) adsorption.

To determine the Pb(II) removal efficiency of the adsorbent, adsorption experiments were performed by adding NE-NFOS into 50 mL of Pb(NO_3_)_2_ solution, and the initial pH value was regulated at 6.0. The mechanical shaker containing the mixture was agitated at 200 rpm at 298 K. After the reaction reached equilibrium, the solution was filtered and the Pb(II) ion concentration was measured using a visible light spectrophotometer (Model 723, Shanghai Jinghua Science & Technology Instrument Co., Ltd, China). The schematic illustration of NE-NFOS preparation and Pb(II) adsorption was shown in Fig. 1. Adsorption was carried out twice, and the average of the two results was used in the discussion of the data. The adsorption capacity at time t, q_t_, can be determined by Equation (1), and the equilibrium adsorption capacity, q_e_, can be determined by Equation (2).

1 $${{\rm{q}}}_{{t}}=\frac{({{C}}_{{o}}-{{C}}_{{t}})\times {\rm{V}}}{{\rm{m}}}$$

2 $${{\rm{q}}}_{{\rm{e}}}=\frac{({{\rm{C}}}_{{\rm{o}}}-{{\rm{C}}}_{{\rm{e}}})\times {\rm{V}}}{{\rm{m}}}$$

where C_0_, C_t_, and C_e_ refer to the concentration of Pb(II) ions at the beginning of the process, at time t, and at equilibrium, respectively, m represents the weight of the NE-NFOS, and V represents the volume of the Pb(II) ion solution.

### Adsorption-desorption recycle experiments

Five recycle experiments were conducted to determine whether the NE-NFOS can be recycled for the removal of Pb(II) ions. For this purpose, 12 mg of the adsorbent was mixed with 20 mL of 50 mg/L Pb(II) ion solution (pH 6.0) for adsorption, and the mixture was agitated at 8000 rpm for 150 min at 298 K. The adsorbent was separated from the Pb(II) ion solution, following which 1.0 M HNO_3_ was used as the regenerant. Then 10 mL of the supernatant was extracted and shaken in a vibrator for 60 min at 200 rpm, and the final Pb(II) ion concentration was determined. Meanwhile, the adsorbent was washed with deionised water for reuse in the subsequent adsorption-desorption cycle.

### Characterization

The morphology of the materials was examined by a FEG250 field-emission scanning electron microscopy (SEM) from Quanta, America. Transmission electron microscopy (TEM) was carried out using a JEM-2100F microscope from JEOL Ltd., Japan. The specific surface area was studied by the Brunauer-Emmett-Teller (BET) method, and the pore size distribution was obtained from the nitrogen adsorption-desorption isotherms using a NOVA 2000e analyser from Quantachrome Instruments, America. Fourier-transformed infrared (FT-IR) spectra were recorded using a VECTOR22 spectrometer from Bruker, Germany.

## Results and discussion

### Adsorbent characterization

**Figure 2:**
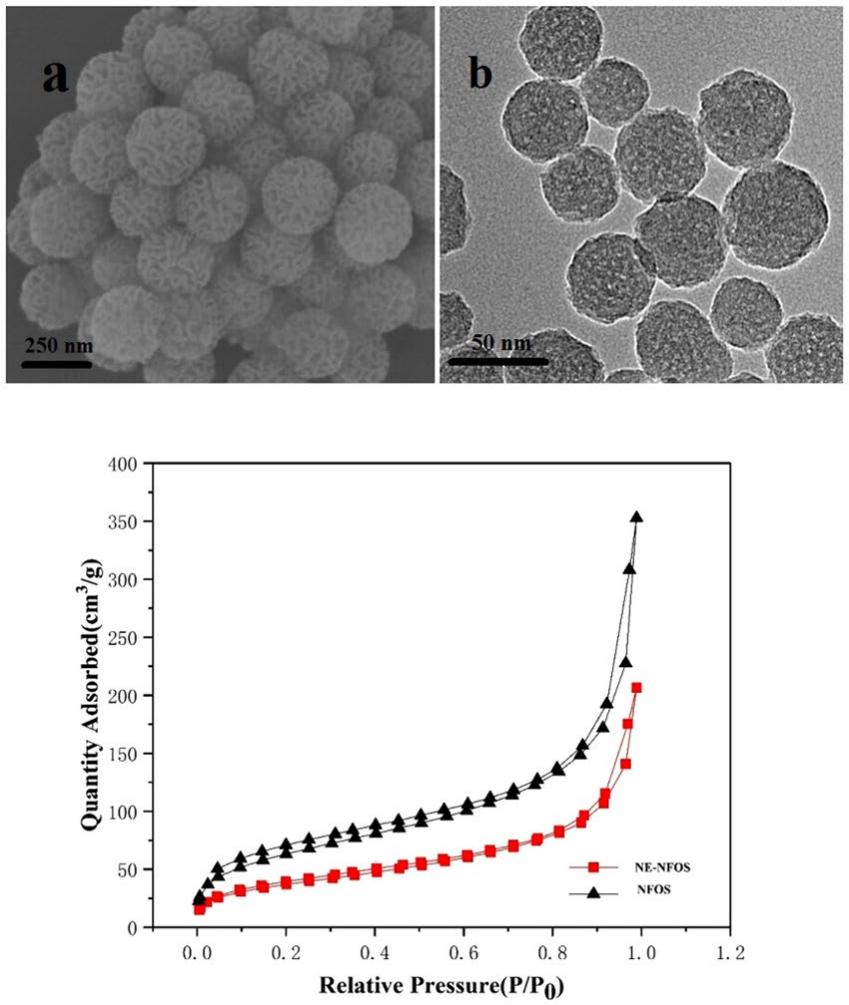
(**a**) SEM image of NE-NFOS, (**b**) TEM image of NE-NFOS, and (**c**) N_2_ adsorption/desorption isotherms.

The SEM image in Fig. 2a indicated that the NE-NFOS had a uniform spherical shape and presented a nanoflower-like structure; its surface was rough and uneven, and the wrinkled channels could increase its surface area, which was beneficial for the adsorption of metal ions^30,31^. Figure 2b presents the TEM image of the NE-NFOS and it could be seen that there were pores in NE-NFOS, and the NE-NFOS was monodisperse particle.

Figure 2c shows the N_2_ adsorption-desorption isotherms of the NFOS and NE-NFOS. It was established that the BET isotherms of the NE-NFOS exhibited characteristic type-IV adsorption-desorption patterns^32^. While the pure NFOS had an average pore size of 3.44 nm and surface area of 236 m^2^/g, the NE-NFOS had a lower pore size of 3.08 nm and smaller surface area of 132 m^2^/g. The pore volume of the NE-NFOS was also reduced to 0.28 cm^3^/g from 0.47 cm^3^/g of the bare NFOS, and this might be attributed to the partial filling of the pores in the NFOS network by the norepinephrine. This phenomenon revealed the success of the modification. Moreover, the large pore size and surface area of NE-NFOS were beneficial for the Pb(II) ions entering the internal pores of the adsorbent, and then improved its sorption capacity.

**Figure 3:**
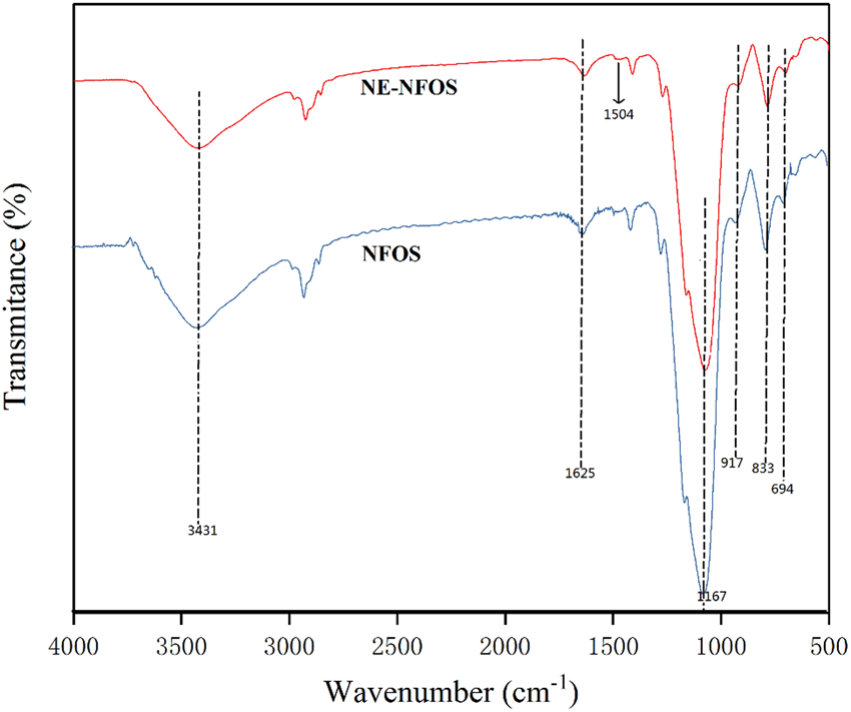
FT-IR spectra of NFOS and NE-NFOS.

Figure 3 presents the FT-IR spectra of the NFOS and NE-NFOS. The absorption bands at 1167 cm^−1^ and 917 cm^−1^ were attributed to the bending vibrations of the Si–O–Si bonds in all the samples^25^. The peaks located at 833 cm^−1^ and 694 cm^−1^ were associated with the symmetric stretching vibrations of the deformation of the Si–OH groups^33,34^. The bands at 3431 cm^−1^ and 1625 cm^−1^ were attributed to the presence of water molecules^35^. Compared to pure NFOS, the characteristic absorption band at around 1504 cm^−1^ was correlated with the benzene ring groups of norepinephrine^35^, suggesting that norepinephrine was grafted on the surface of the NFOS.

### Adsorption kinetics

**Figure 4:**
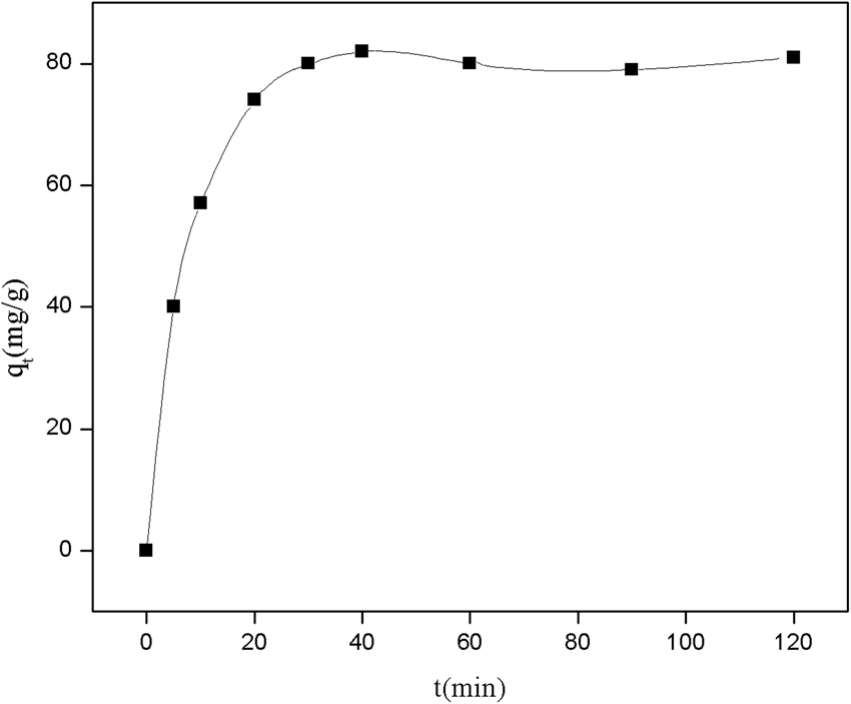
Influence of contact time on Pb(II) ion adsorption.

The influence of contact time between the adsorbent and Pb(II) ions was studied to measure the adsorption kinetics. The adsorption experiments were carried out by adding the adsorbent into a lead solution (pH 6.0) with initial Pb(II) ion concentration of 100 mg/L. The results, presented in Fig. 4, established that the adsorption capacity improved greatly with increase in contact time. The adsorption proceeded in three stages. In the first stage from 0 to 20 min, the adsorption proceeded rapidly, whereas in the second stage from 20 to 40 min, the process progressed gradually, and adsorption equilibrium was eventually attained. In the third stage from 40 to 120 min, the adsorption amount of Pb(II) on the NE-NFOS did not increased.

The initial rapid adsorption in the first stage was due to the fact that the adsorbent dispersed well and quickly in the aqueous solution, and the concentration gradient of Pb(II) ions was larger. Additionally, the surface adsorption sites of NE-NFOS were abundant, and they were occupied by Pb(II) ions rapidly. Subsequently, penetration resistance increased and more energy was required, and this led to a rapid decrease in the adsorption rate in the second stage of the process^36^. Adsorption equilibrium for Pb(II) ion removal was achieved in less than 40 min, and hence a duration of 90 min was selected to ensure complete removal of Pb(II) ions.

### Adsorption isotherms

**Figure 5:**
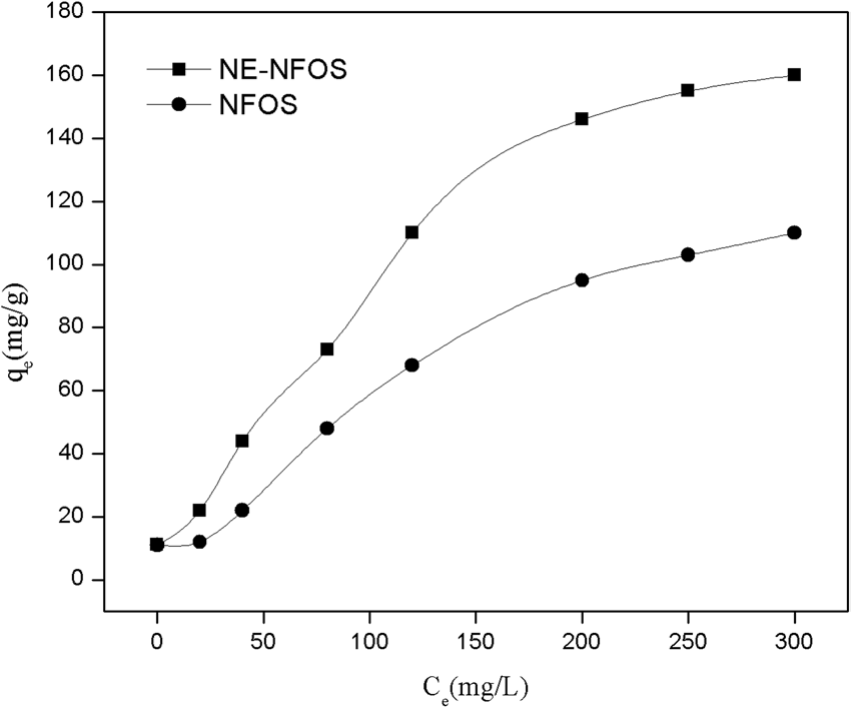
Adsorption isotherms of Pb(II) ions adsorbed on to NE-NFOS and NFOS.

Adsorption isotherms were used to inspect the detailed adsorption characteristics. The experiments were carried out with Pb(II) ion concentration in the range 20–300 mg/L; the NE-NFOS was added into the Pb(II) ion solution (pH 6.0) at 298 K. The results are presented in Fig. 5.

The adsorption capacity of the NE-NFOS improved with increase in Pb(II) ion equilibrium concentration. The maximum adsorption capacity was 160 mg/g, which was mainly attributed to the fact that the functional groups on the surface of the NE-NFOS had a favourable ability of chelation with Pb(II) ions after modification^28^. Because of Pb(II) belonging to borderline metal, which has ambivalent property, the amino groups in the molecules of norepinephrine have favourable affinity with Pb(II)^22,23^. Moreover, the phenolic groups in norepinephrine molecules have favorable bidentate chelating ability with a variety of heavy metal ions^37^, hence, the two oxygen atoms in the phenolic groups could bound to a Pb(II) ion, and the adsorption capacity of NE-NFOS for Pb(II) ions was improved.

Additionally, the Langmuir and Freundlich adsorption isotherm equations were used to describe the adsorption characteristics of the NE-NFOS for Pb(II) ion removal^38^, and their linear equations are shown in Equations (3) and (4):

3 $$\frac{{{\rm{C}}}_{{\rm{e}}}}{{{\rm{q}}}_{{\rm{e}}}}=\frac{{\rm{1}}}{{{\rm{q}}}_{{\rm{m}}}{{\rm{K}}}_{{\rm{L}}}}+\frac{{{\rm{C}}}_{{\rm{e}}}}{{{\rm{q}}}_{{\rm{m}}}}$$

4 $$\mathrm{ln}\,{{\rm{q}}}_{{\rm{e}}}=\,\mathrm{ln}\,{{\rm{K}}}_{{\rm{f}}}+\frac{{\rm{1}}}{{\rm{n}}}\,\mathrm{ln}\,{{\rm{C}}}_{{\rm{e}}}$$

Here, q_e_ represents the adsorbent’s capacity for Pb(II) ion removal; C_e_ represents the solution concentration; q_m_ refers to the maximum adsorption capacity of the NE-NFOS for Pb(II) ion removal; K_f_ and n are constants of the Freundlich model.

**Figure 6:**
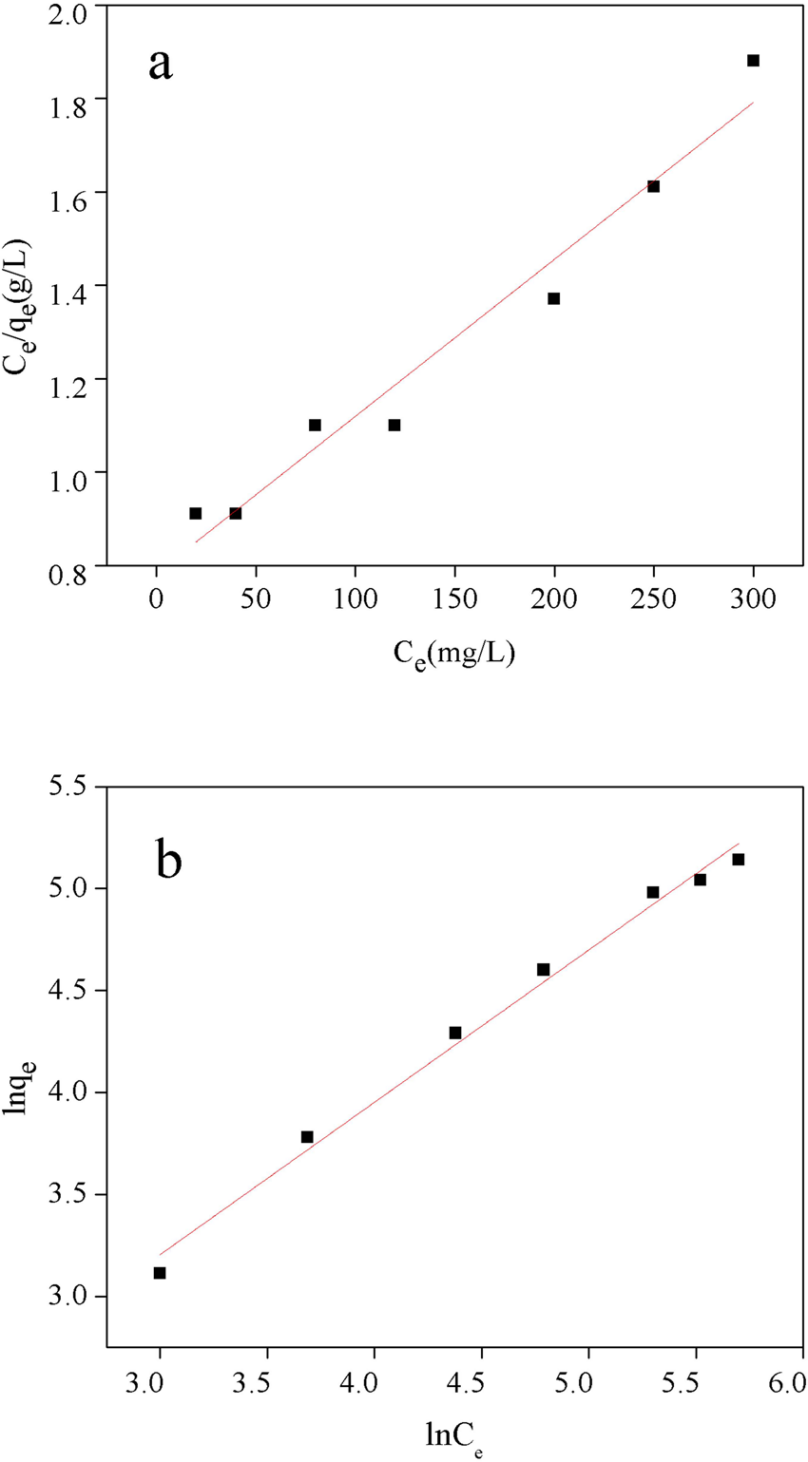
Linearised (**a**) Langmuir model plot (Eq. (3)) and, (**b**) Freundlich model plot (Eq. (4)) for Pb(II) adsorption by the NE-NFOS.

A comparison of the Langmuir and Freundlich isotherm parameters is shown in Table 1, while Fig. 6 presents the fitted curves of the two models. The correlation coefficients indicated that the adsorption was fitted better by the Freundlich model (R^2^ = 0.991) than the Langmuir model (R^2^ = 0.965), as shown in Fig. 6. Therefore, the process could be explained by multilayer adsorption and the heterogeneous system^39^.

**Table 1:** Adsorption isotherm parameters for Pb(II) ion removal.

Model Parameters	Langmuir			Freundlich		
	K_L_	q_m_ (mg/g)	R^2^	K_F_	q_m_ (mg/g)	R^2^
Value	0.004	294	0.965	2.620	186	0.991

### Influence of pH

**Figure 7:**
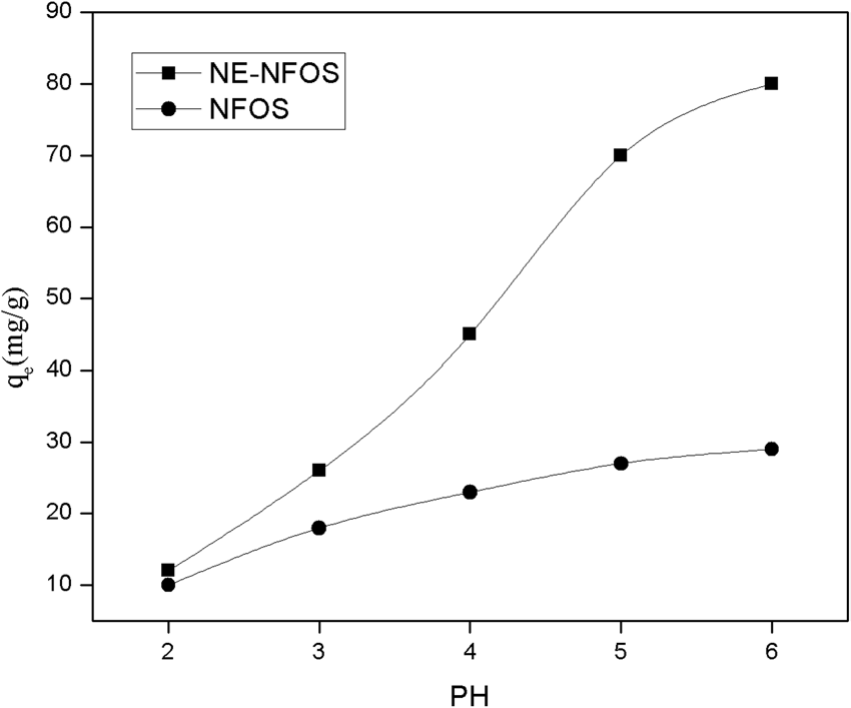
Effect of solution pH on Pb(II) ion removal.

Figure 7 presents the adsorption ability of the NE-NFOS for removing Pb(II) ions from aqueous solutions of different pH values^40^. The studies were conducted at 298 K, with pH in the range 2–6, and adsorbent dosage of 0.8 g/L. The pH values were adjusted with HNO_3_ or NaOH solution. The adsorption capacity was found to increase as pH rose from 2 to 6. At lower values of solution pH, the adsorption capacity was small, which could partly be attributed to competition between the Pb(II) and H^+^ ions for the adsorption sites on the mesoporous silica^41^. Another reason was that the surface of the adsorbent was positively charged and exhibited electrostatic repulsion towards the Pb(II) ions, resulting in few active sites being available for Pb(II) adsorption^18,42^. Hence, the efficiency of Pb(II) ion removal by the NE-NFOS was restricted. At higher values of solution pH, an increasing number of H^+^ ions left the surface of the adsorbent, making more adsorption sites available for the Pb(II) ions. Additionally, at higher solution pH, the NE-NFOS had a highly negative surface charge and exhibited stronger chelating ability for metal ions, which resulted in significant improvement in its adsorption capacity.

### Effect of Na^+^, K^+^, Ca^2+^, and Mg^2+^ concentration on Pb(II) ion adsorption

**Figure 8:**
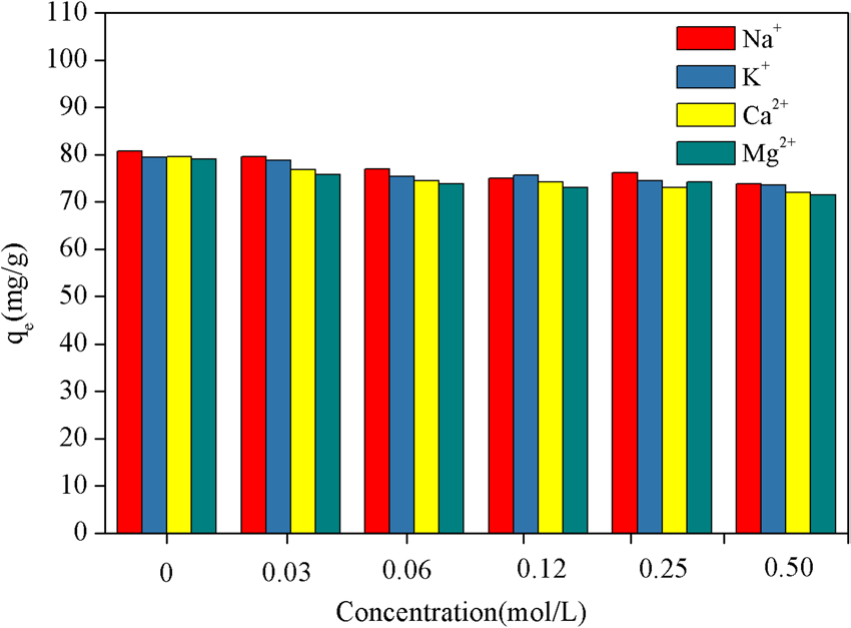
Effect of Na^+^, K^+^, Ca^2+^ and Mg^2+^ concentrations on Pb(II) ion adsorption.

To investigate the competitive adsorption of coexisting ions on to the binding sites, the adsorption experiments were carried out on solutions mixed with different metal ions, in the presence of KNO_3_, NaNO_3_, CaCl_2_, and MgCl_2_, respectively. The NE-NFOS was mixed with a Pb(II) ion solution with initial concentration of 103.6 mg/L at 298 K and pH of 6.0. The concentration of the metal ions was in the range 0–0.5 mol/L, and the solutions were shaken for 90 min. The results, shown in Fig. 8, revealed that the concentrations of Na^+^, K^+^, Ca^2+^, and Mg^2+^ had a slight effect on the adsorption of Pb(II) ions. This could be attributed to the fact that the functional groups on the surface of the NE-NFOS had more powerful chelation capability than did the Na^+^, K^+^, Ca^2+^ and Mg^2+^ ions. Moreover, the slight decline in the Pb(II) ion adsorption capacity was likely a result of competition between the Pb(II) and other metal ions^43^.

### Desorption and reusability

**Figure 9:**
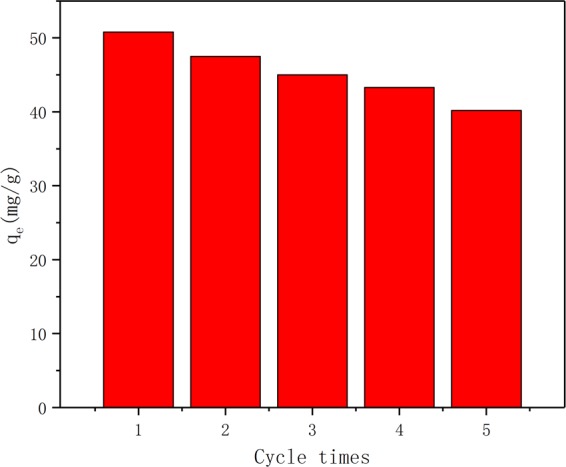
Reusability of the NE-NFOS for five cycles.

To investigate the desorption and reusability of the NE-NFOS for Pb(II) ion removal, five adsorption-desorption cycles were conducted. These were carried out by adding 60 mg of the NE-NFOS into 50 mL of Pb(II) ion solution with initial concentration of 50 mg/L and pH of 6.0 at 298 K. The mixture was then shaken for 90 min. The results are listed in Fig. 9. It was evident that after five adsorption-desorption cycles, the adsorption capacity of the NE-NFOS exceeded 79% of its initial value, indicating that the NE-NFOS had favourable reusability. Therefore, the NE-NFOS could not only be used as a recyclable adsorbent for the removal of Pb(II) ions from aqueous solutions, but also the Pb(II) ions could be recovered as precious resources. Hence, we envision that this efficient and recyclable adsorbent of NE-NFOS had great potential for the practical applications in the removal and recovery of Pb(II) ions from waste water.

### Adsorption of Cu(II), Cd(II) and Pb(II) ion on NE-NFOS

**Figure 10:**
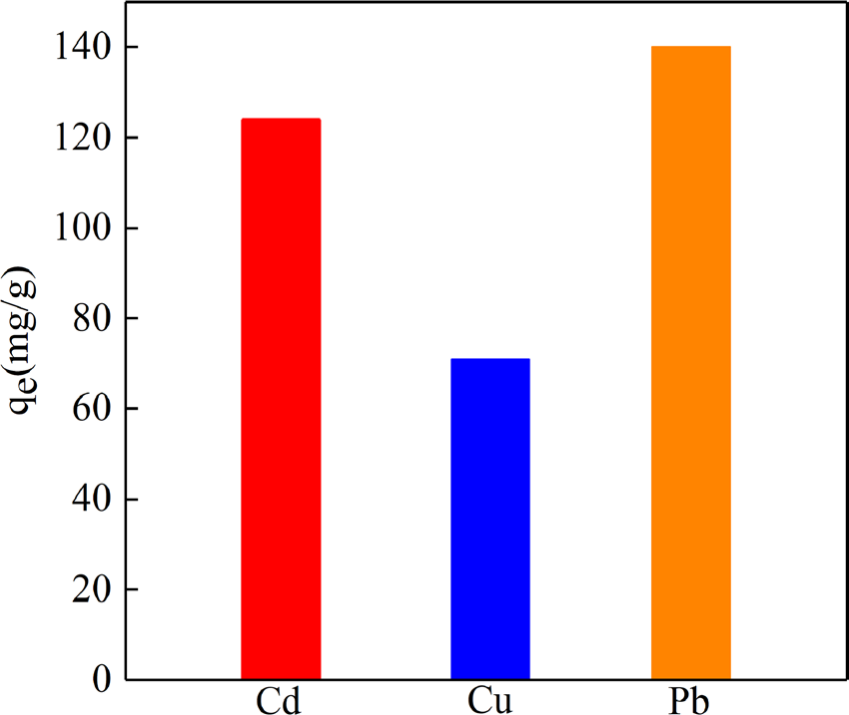
Adsorption of Cu(II), Cd(II) and Pb(II) ions on NE-NFOS.

The phenolic groups and amino groups in the molecules of norepinephrine have favourable affinity with a variety of heavy metal ions^37^, therefore, it was anticipated that the NE-NFOS could exhibit high performance in the adsorption of other heavy metal ions. Hence, the adsorption capacities of Cu(II) and Cd(II) ions on NE-NFOS were studied, and the experiments were carried out by adding 60 mg of the NE-NFOS into 50 mL of Cu(II) or Cd(II) ions solution with initial concentration of 200 mg/L and pH of 6.0 at 298 K, respectively. For comparison, the adsorption experiments of Cd(II) by NE-NFOS were carried out at the same conditions, and the results are shown in Fig. 10. According to the results, the adsorption amounts of Cd(II), Cu(II) and Pb(II) on the NE-NFOS were 124 mg/g, 71 mg/g and 140 mg/g, respectively, which demonstrated that the NE-NFOS possessed great potential in purifying a variety of heavy metal ions from waste water.

### Conclusions

Norepinephrine-functionalised mesoporous adsorbent was developed for the removal of Pb(II) ions from aqueous solutions. Characterization of the NE-NFOS using techniques including SEM, TEM, BET, and FT-IR indicated that norepinephrine had successfully modified the surface of the NFOS. The adsorption capacity of the NE-NFOS for Pb(II) ions removal reached as high as 160 mg/g and the adsorption process could be described by the Freundlich adsorption equation. Additionally, the presence of Na^+^, K^+^, Ca^2+^ and Mg^2+^ ions had a very weak influence on the removal of Pb(II) ions by the NE-NFOS. The adsorption-desorption cycle experiments indicated that the NE-NFOS could retain 79% of its initial adsorption capacity even after it was recycled five times. Moreover, the NE-NFOS exhibited favourable adsorption capacity for the Cd(II) and Cu(II) ions. Thus, it could be concluded that the NE-NFOS had great potential as an effective adsorbent to remove heavy metal ions from aqueous solutions.
